# Fabrication of Hydrogels with Steep Stiffness Gradients for Studying Cell Mechanical Response

**DOI:** 10.1371/journal.pone.0046107

**Published:** 2012-10-04

**Authors:** Raimon Sunyer, Albert J. Jin, Ralph Nossal, Dan L. Sackett

**Affiliations:** 1 Program in Physical Biology, Eunice Kennedy Shriver National Institute of Child Health and Human Development, National Institutes of Health, Bethesda, Maryland, United States of America; 2 Laboratory of Cellular Imaging and Macromolecular Biophysics, National Institute of Biomedical Imaging and Bioengineering, National Institutes of Health, Bethesda, Maryland, United States of America; The University of Akron, United States of America

## Abstract

Many fundamental cell processes, such as angiogenesis, neurogenesis and cancer metastasis, are thought to be modulated by extracellular matrix stiffness. Thus, the availability of matrix substrates having well-defined stiffness profiles can be of great importance in biophysical studies of cell-substrate interaction. Here, we present a method to fabricate biocompatible hydrogels with a well defined and linear stiffness gradient. This method, involving the photopolymerization of films by progressively uncovering an acrylamide/bis-acrylamide solution initially covered with an opaque mask, can be easily implemented with common lab equipment. It produces linear stiffness gradients of at least 115 kPa/mm, extending from ∼1 kPa to 240 kPa (in units of Young's modulus). Hydrogels with less steep gradients and narrower stiffness ranges can easily be produced. The hydrogels can be covalently functionalized with uniform coatings of proteins that promote cell adhesion. Cell spreading on these hydrogels linearly correlates with hydrogel stiffness, indicating that this technique effectively modifies the mechanical environment of living cells. This technique provides a simple approach that produces steeper gradients, wider rigidity ranges, and more accurate profiles than current methods.

## Introduction

The mechanical properties of the extracellular matrix (ECM) contribute to the regulation of many important cell processes that determine cell fate and function [1]. Examples of cellular functions regulated by mechanical cues include cell proliferation, migration, spreading, morphology and the differentiation of stem cells [2]–[4]. Most studies in these areas have focused on how cells respond to a substrate of uniform stiffness. However, the stiffness of cell microenvironment displays high variation within the body. Between different tissues, extracellular matrix rigidity often varies over several orders of magnitude, e.g., brain (260–490 Pa), liver (640 Pa), kidney (2.5 kPa), skeletal muscle (12–100 kPa) and cartilage (950 kPa) (Reviewed in Ref. [5]). Moreover, local stiffness can vary strongly, giving rise to complex rigidity gradients that can span several orders of magnitude, such as those noted at interfacial tissues [6]. Tissue variation can also be caused by pathological factors such as malignant tumors, which are stiffer than the healthy tissue that surrounds them [7], [8]. Stiffness differences play a crucial role, for instance, in the directed migration of fibroblasts which move from soft to stiff regions of the ECM. This process often is referred to as “durotaxis” or “mechanotaxis” [9]. Mesenchymal stem cells differentiate after undergoing durotaxis and their lineage specification is modulated, not only by average matrix stiffness, but by stiffness variation as well [10]. Finally, stiffness gradients have been suggested to be important cues guiding the migration of cancer cells in the interstitial ECM towards sites of intravasation [8].

In order to study these processes, it is necessary to develop methods to fabricate defined-stiffness gradient profiles on hydrogel substrates to which ECM components are covalently bound. One commonly used scheme is to vary the crosslink density of a polyacrylamide (PAA) hydrogel which underlies the surface upon which cells are deposited. The crosslink density has often been modulated by varying the ratio between acrylamide and the crosslinker bis-acrylamide, and initiating the reaction with the soluble initiator TEMED [11]. By placing two droplets -one containing a soft and the other a stiff acrylamide/bis-acrylamide mixture- adjacent to each other and covering them with a common coverglass, a rudimentary, poorly defined, stiffness gradient can be created [9]. An alternative technique involves photoinitiated polymerization of the acrylamide/bis-acrylamide solution. With this method, the crosslink density of the hydrogel depends on the amount of light that the hydrogel receives, which can be adjusted by using a variable gray-level photomask [12]. Although the simplicity of this method makes it easy to implement, the low resolution of the mask severely limits the precise control of the gradient profile at the micrometer scale. To overcome this limitation, microfluidic gradient generators have been used to combine varying amounts of acrylamide/bis-acrylamide solution in a single hydrogel [13], [14]. Even though this technique is able to produce matrices with steeper gradient, the rigidity range is again limited. Moreover, this technique is costly, time consuming, and linear stiffness profiles are difficult to implement with precision.

Here, we introduce a new method which produces inexpensive, high gradient matrices whose stiffness profile at the micrometer scale can be tightly controlled. This method allows fabrication of well defined stiffness profiles in PAA hydrogel matrices. Subsequent addition of a layer of ECM protein is unaffected by the varying stiffness of the underlying hydrogel. This method can be easily implemented with common lab equipment and produces stiffness gradients and rigidity ranges higher than the ones prepared with microfluidic devices, allowing for precise control of the gradient profile at the micrometer scale. Although the scheme described here has more general applicability, we focus on PAA hydrogels because they have been widely used as a matrix support for cells [11], [15], [16], have well documented mechanical properties [17], and are amenable to covalent coating with ECM proteins [16]. We demonstrate that this method is suitable for studying the mechanical response of cells to substrates of different stiffness.

## Results

**Figure 1:**
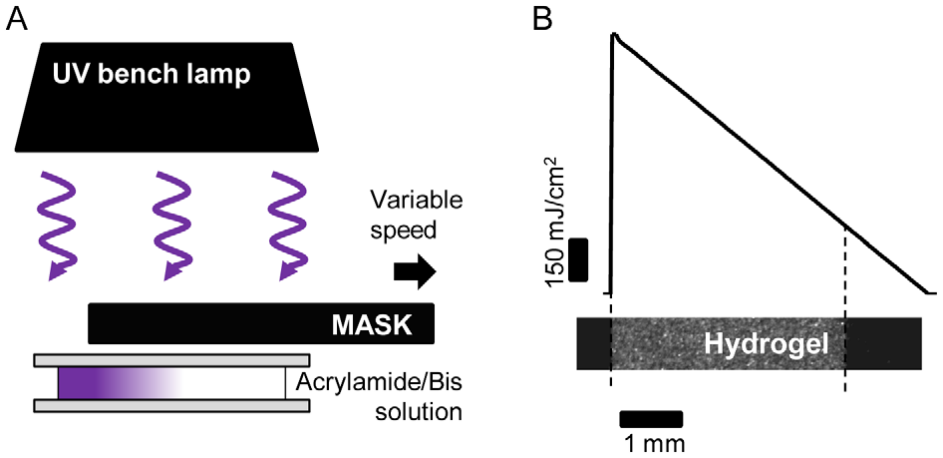
Sketch of the experimental setup. (A) To produce hydrogels with a stiffness gradient, we irradiate an acrylamide/bis-acrylamide solution, containing Irgacure, confined between two coverslips of ∼170 µm thickness (#1). The sample initially is protected by an opaque mask. An irradiation gradient is obtained by moving the mask (in the direction of the arrow) while illuminating the solution with a non-collimated UV lamp (365 nm). (B) Linear irradiation gradient obtained by moving the opaque mask at a constant speed. Any arbitrary, monotonically decreasing irradiation pattern can be generated by changing the mask speed during the polymerization process.

Stiffness gradient hydrogels were obtained by irradiating an acrylamide/bis-acrylamide solution (containing the photoactivatable initiator Irgacure) with a varying dose of light, achieved by covering the solution with an opaque mask and then moving the mask at controlled speed to progressively uncover the gel solution (Fig. 1A, see Methods). In this way, we were able to obtain a well-defined irradiation pattern (Fig. 1B). The edge of the mask defined the boundary between darkness and light, thereby creating a profile of monotonically decreasing total irradiation. To calibrate our system, we first moved the mask at constant speed (15 µm/sec), producing a linear irradiation profile as typified by the plot shown in Fig. 1B.

**Figure 2:**
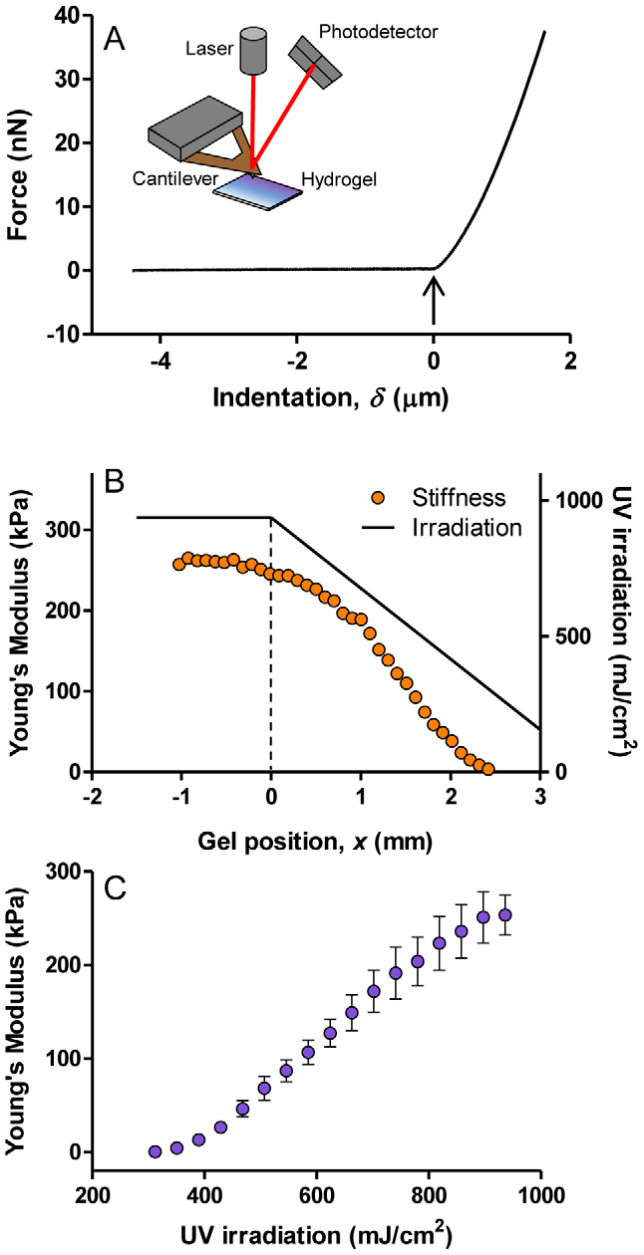
Characterization of the stiffness gradient hydrogels. (A) Schematic of AFM hydrogel mechanical measurement: (Cartoon) Illustration of an AFM probing the hydrogel elastic modulus; (Graph) Force–indentation (*F*–*δ*) curve obtained as the cantilever tip approached and indented a hydrogel (solid line) at 10 µm s^−1^.This curve was used to determine the contact point between the tip and the sample (arrow). The rising portion of the curve yielded the Young's modulus (*E*) of the hydrogel. (B) Hydrogel stiffness variation (circles) produced by a linear irradiation profile (solid line). At the beginning of the fabrication process, the opaque mask is placed at gel position *x* = 0 mm (indicated by a dashed line). For *x*<0, the hydrogel irradiation (solid line) is constant during the fabrication process. As the fabrication begins, the mask is moved at a constant speed of 15 µm s^−1^. This results in a decreasing irradiation from the dashed line to the end of the gel. Although the irradiation has a linear profile, the Young's modulus of this hydrogel region decreases non-linearly from 240 kPa to ∼1 kPa. (C) Hydrogel stiffness as a function of irradiation dose. We use this calibration curve to compute the mask speed profile needed to linearize the gel stiffness profile (see Text S1, Fig. S2 and Fig. S3). Error bars represent SE of 4 replicates.

Thin PAA hydrogels polymerized in this manner were characterized with Atomic Force Microscopy (AFM). An example of a force-indentation curve recorded from the hydrogel is shown in Fig. 2A. For indentations *δ*<0, the cantilever was not in contact with the hydrogel. As the cantilever pyramidal tip contacted the hydrogel at *δ* = 0 (arrow), the force recorded by the cantilever started to increase, exhibiting a non-linear relationship with the indentation. This non-linear relationship is caused by an increase of the contact area as the pyramidal tip indents the sample. By fitting the contact part of the force-indentation curve to the 4-sided pyramidal indenter model [18], we precisely determined the Young's modulus (*E*) as a function of position on the gel surface.

### Fabrication of linear stiffness hydrogels

Figure 2B illustrates the spatial variation of the Young's modulus, *E*, of a hydrogel produced by a linear UV irradiation profile. At the beginning of the fabrication process, the opaque mask was placed at gel position *x* = 0 mm (indicated by a dashed line in Fig. 2B), covering the area from *x* = 0 to *x* = 3.0 mm. The irradiation of the gel region *x*<0 mm was unchanged during the fabrication process (Fig. 2B black line). The Young's modulus (Fig. 2B, circles) in this region was approximately constant up to *x* = 0 mm. When the mask was moved at a constant speed of 15 µm/s, the irradiation continuously decreased between 0 mm and the end of the hydrogel (*x* = 2.4 mm). Depending on the mask speed, we obtained different gradient slopes while maintaining the same rigidity range (Fig. S1). Although the irradiation had a linear profile, the Young's modulus of this region decreased non-linearly from 240 kPa to ∼1 kPa.

**Figure 3:**
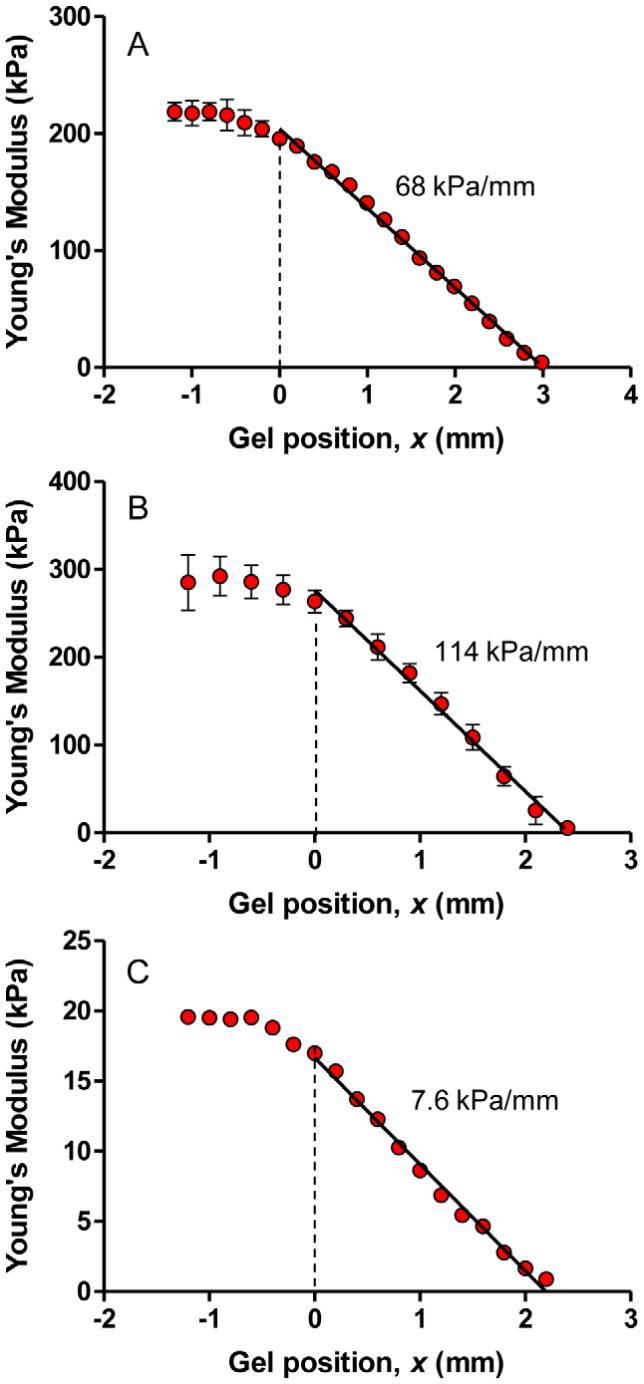
Profile of the elasticity of hydrogels produced by linearizing the stiffness gradient as explained in Text S1, Fig. S2 and Fig. S3. (A) A solution of 15% acrylamide and 1% bis-acrylamide produces a stiffness gradient hydrogel of ∼68 kPa/mm when irradiated for 240 s with the speed protocol optimized to linearize the stiffness profile (error bars: SD of 3 replicates). (B) The same acrylamide/bis-acrylamide solution produces a stiffness gradient hydrogel of ∼114 kPa/mm when illuminated for 270 s with a similar variable speed protocol (error bars: SD of 6 replicates). (C) A shallower stiffness gradients of ∼7.5 kPa/mm can be obtained by using a solution of 8% acrylamide and 0.48% bis-acrylamide and irradiating for 240 s with a variable speed protocol (Mean of 2 replicates). Note that panels A–C share an almost identical scale in the *x*-axis but different scales in the *y*-axis.

To linearize the stiffness gradient of the hydrogel, we first measured the Young's modulus of different hydrogels as a function of the irradiation exposure time, observing a non-linear monotonic increase of the Young's modulus when plotted against the irradiation (Fig. 2C). Using this calibration curve, we calculated the speed protocol that linearizes the stiffness gradient profile (see Text S1, Fig. S2 and Fig. S3). We obtained a 3 mm long hydrogel whose Young's modulus decreased linearly from 200 kPa to ∼1 kPa, with a stiffness gradient of ∼68 kPa/mm (Fig. 3A). Hydrogel stiffness profile was reproducible; the main sources of variability were pipetting errors and Irgacure preparation (see Fig. S4). The stiffness gradient of these hydrogels is much greater than those obtained with other techniques such as photopolymerization modulated by gray-intensity masks [10], [12] and microfluidics [13], [14]. Using different mask speed protocols and different bis-acrylamide solutions, we are able to produce other linear gradients, as exemplified by Figs. 3B and 3C.

### Substrate functionalization

**Figure 4:**
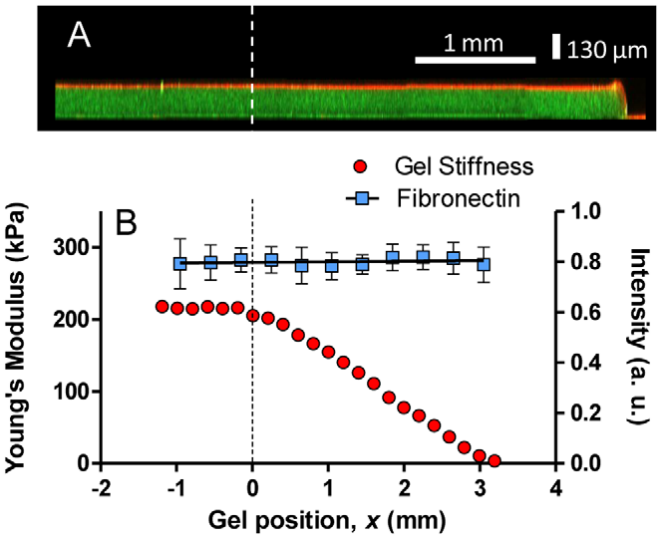
Fibronectin density is independent of gel stiffness. (A) Confocal cross-sectional fluorescence image of a stiffness gradient hydrogel confirms that the protein functionalization (fibronectin, stained in red) is confined to the top surface of the hydrogel and that the hydrogel (marked with embedded green fluorescent beads) thickness is constant across the different stiffness areas. Dashed line indicates initial position of the mask. The left edge of the hydrogel has been truncated. (B) Fluorescence intensity profile along a gradient hydrogel coated with fibronectin and labeled with antibody (squares) is shown together with spatial variation of hydrogel elasticity (circles). Error bars represent SE of 3 replicates.

To promote cell adhesion, fibronectin was covalently linked to the PAA hydrogels via sulfo-SANPAH mediated succinimide cross-linking. We used immunofluorescence against fibronectin to confirm that the density of protein on the gel surface does not spatially vary with the stiffness of the underlying matrix. Because amines can be highly adsorptive, we performed an experimental control to check that the fibronectin was incorporated on the hydrogel surface by succinimide chemistry rather than nonspecific associations. We quantified the intensity of the immuofluorescence across the hydrogel and observed that after fibronectin incubation, hydrogels treated with sulfo-SANPAH incorporate substantially more fibronectin than untreated ones (see Fig. S5). Confocal cross-sectional fluorescence images of the hydrogels confirm that the protein functionalization is confined to the top surface and that hydrogel thickness is constant across different stiffness areas (Fig. 4A). As mentioned above, the stiffness of the tested hydrogels shows different regions. In the example shown, for *x*<0 mm the hydrogel displays a plateau of approximately 225 kPa (Fig. 4B). For *x*>0 mm, the Young's modulus of the hydrogel linearly decreases from 225 kPa to ∼1 kPa. Quantification of the immuofluorescence across the entire gel indicates that the fluorescence associated with the fibronectin does not change as the hydrogel stiffness varies (Fig. 4B). Consequently, the gradient in stiffness does not induce a gradient in the protein ligand density.

### Quantitative analysis of cell response to substrate stiffness

**Figure 5:**
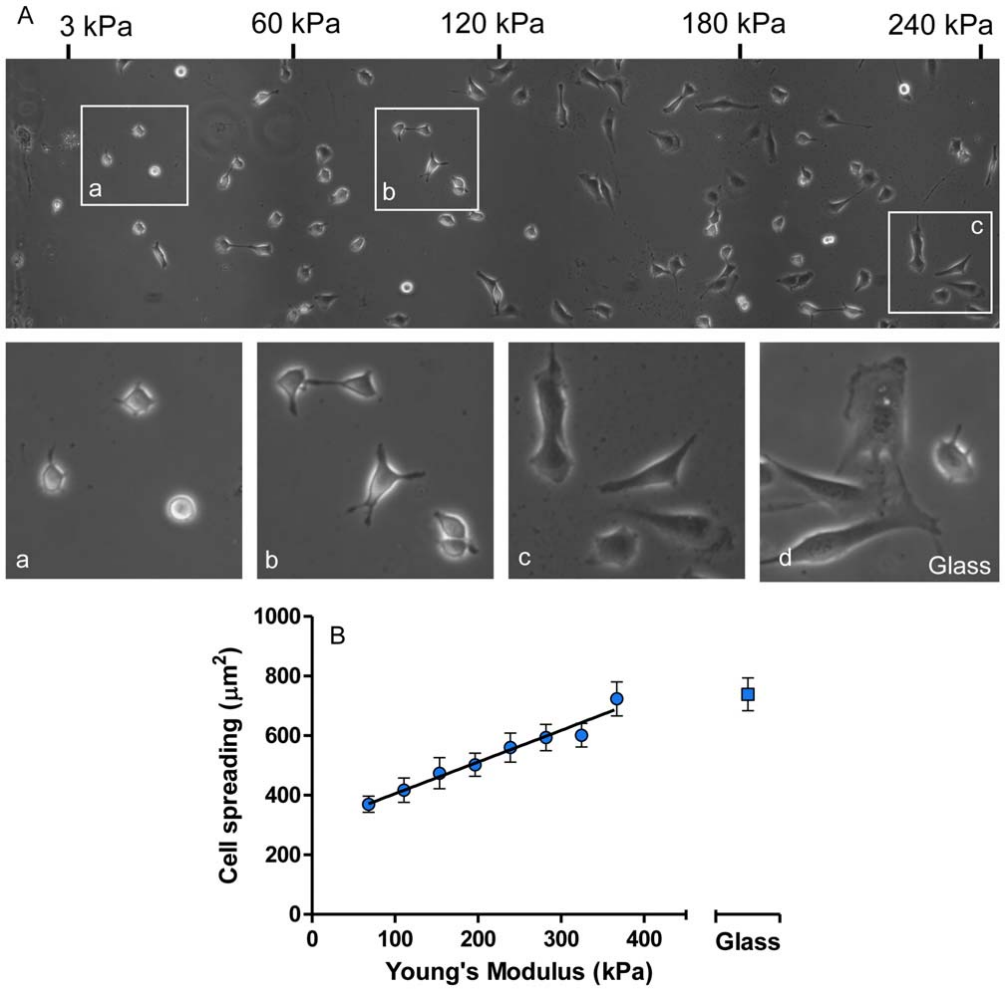
Spreading of cells correlates with hydrogel stiffness. (A) Phase contrast image of NIH3T3 fibroblasts on a stiffness gradient hydrogel functionalized with fibronectin. The hydrogel stiffness increases towards the right of the image. Numbers on the top indicate Young's modulus values. Individual panels (a–c) qualitatively show that cell spreading increases with hydrogel stiffness. Panel (d) is an image of spreading cells on glass. (B) Quantitative analysis of cell spreading as a function of hydrogel stiffness. The spreading of cells attached to the stiffer part of the hydrogel, compared with the spreading of cells on glass. Error bars represent SE.

We seeded early passage fibroblasts (NIH3T3 cell line) on our stiffness gradient hydrogels. To maximize the stiffness range accessible per field of view, we used a hydrogel whose stiffness varied linearly from ∼1 kPa to 240 kPa across 2 mm and then non-linearly to 360 kPa across an additional 2 mm. Phase contrast images reveal that cell spreading strongly correlates with hydrogel stiffness (Fig. 5A, and panels a–c). Cells located at the softer part of the hydrogel display a rounded morphology and poor spreading (Fig. 5A, panel a). Conversely, cells on the stiffer part of the hydrogel or on glass appear to be well spread and well adhered (Fig. 5A, panel c and d). When quantifying NIH3T3 cell spreading area, we observed that early passage cells present smaller spreading than cells maintained for longer times. The spreading of both early and later passage NIH3T3 cells directly correlated with stiffness allowing us to quantify cell response to substrate stiffness by fitting a linear model to our data (Fig. 5B, black line). For early passage NIH3T3 cells, we found that spreading varied as 9.6±0.75 µm^2^/kPa. For comparison, neuroblastoma cells (SY5Y cell line) display a similar correlation between spreading and stiffness with a slightly higher stiffness sensitivity (16.2±2.1 µm^2^/kPa, data not shown). Notably, cells located at the stiffer part of the hydrogel had a spreading comparable to cells seeded on glass when also coated with fibronectin.

**Figure 6:**
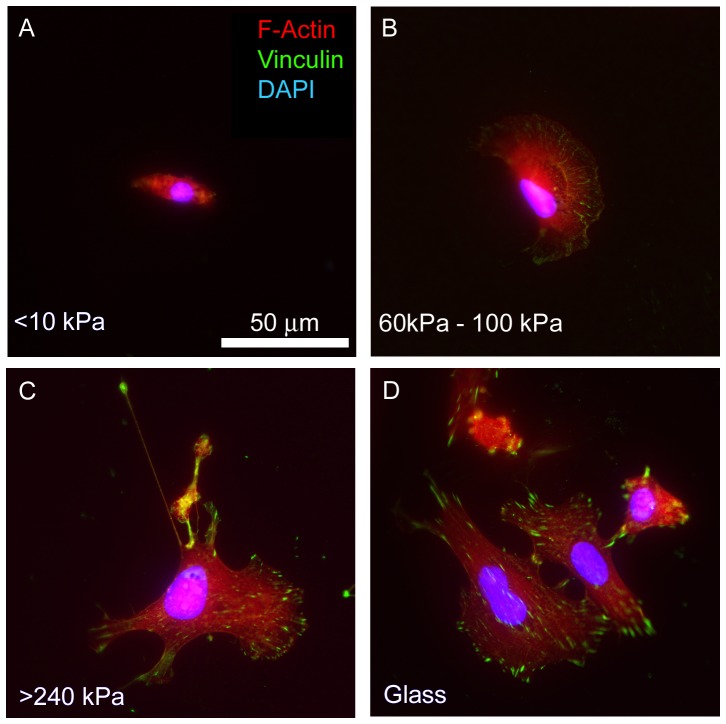
Cytoskeletal organization changes as hydrogel rigidity is altered. Representative fluorescent images of NIH3T3 cells stained for F-actin (red), focal adhesion protein vinculin (green), and nuclear DNA (blue, although appears slightly purple due to the actin colocalization). Panels A–C show cell patterns obtained on different stiffness regions on the same hydrogel. Panel D shows cells on glass coated with fibronectin.

Changes in cell spreading correlate with changes in cytoskeleton organization (Fig. 6). Cells located on the softer part of the hydrogel (<10 kPa) do not show stress fibers and focal adhesions (Fig. 6A). As the hydrogel becomes stiffer, the actin cytoskeleton becomes more highly organized and focal adhesions start to appear (Fig. 6B). At stiffer end of the hydrogel (Fig. 6C), the cells show stress fibers and focal adhesion comparable to those of cells attached to glass (Fig. 6D). It has been extensively documented that cell spreading and cytoskeleton organization vary with substrate rigidity [2], [3], [19]. Our method for fabricating stiffness gradient matrices allows these measurements to be performed with a single hydrogel containing a broad rigidity range.

## Discussion

We have developed a new method to fabricate hydrogels having well defined, linear stiffness gradients. AFM reveals that our technique produces stiffness gradients up to 115 kPa/mm, with a rigidity range that varies linearly from ∼1 kPa to 240 kPa, followed by a region of increasing but non-linear stiffness change in the 240–360 kPa range. Fabrication of gradients is difficult in this upper range which is near the limit of stiffness possible with this material. Hydrogels with less steep gradients and narrower stiffness ranges can easily be produced as well. The hydrogels can be covalently functionalized with proteins that promote cell adhesion. Immunofluorescense against fibronectin reveals that the ligand density is independent of the stiffness gradient. The spreading of cells attached to stiffness gradient hydrogels correlates linearly with hydrogel rigidity. Cell spreading strongly differs in regions with different rigidity, indicating that hydrogels produced with this technique effectively mimic the mechanical environment of living cells.

The method presented here utilizes the photoinitiator Irgacure 2959 to fabricate elasticity gradients in polyacrylamide hydrogels. When illuminated with UV light, Irgacure molecules decompose into free radicals that initiate polyacrylamide polymerization [20]. Similar to the polymerization initiated by TEMED and ammonium persulfate, the released free radicals initiate the polymerization of the acrylamide and the bis-acrylamide crosslinker. By modulating the amount of UV light, we can control the amount of acrylamide chains that are crosslinked by the bis-acrylamide. By systematically and spatially varying the dose of UV light delivered to the solution a gradient of elasticity is created.

Other methods have been developed to obtain PAA hydrogels with a stiffness gradient that covers part of the physiological range. As mentioned earlier, the simplest technique is to place two droplets having differing concentrations of acrylamide/bis-acrylamide next to each other on a common coverslip [9]. Using this approach, a non-linear and undefined stiffness profile can be obtained in the range of stiffness accessible to PAA hydrogels. A somewhat more precise method involves photopolymerization modulated by a fixed gray-intensity mask. This scheme produces hydrogels with better defined stiffness profiles, but the low resolution of the masks limits these gels to a gradient of ∼1 kPa/mm and a rigidity range of 1–14 kPa [10], [12]. Microfluidic gradient generators substantially improve the rigidity profile. In its most extended version, varying amounts of photocrosslinkable acrylamide/bis-acrylamide solution are combined in a single channel and polymerized under UV irradiation [3], [14]. This technique produces a non-linear gradient in the 3–40 kPa range with an average gradient of 14 kPa/mm [14]. An improved stiffness profile (up to 40 kPa/mm) and a higher rigidity range (3–80 kPa) is obtained by increasing the amount of acrylamide in the solution [13].

By contrast, our method, allows us to create accurate linear profiles with a 3-fold steeper gradient and a 4.5-fold larger rigidity range than those obtained with microfluidic gradient generators. In contrast to hydrogels produced with microfluidic techniques, our method does not need access to clean room facilities and costly equipment. In addition, the fabrication process is faster, simpler, and might be extended to 3D cell culture. To our knowledge, our fabrication method is the easiest and most precise way to obtain hydrogels with a high stiffness gradient and a broad rigidity range.

The idea of moving an opaque mask to produce an irradiation gradient was initially used to characterize the photopolymerization kinetics of industrial polymers [21]. In the biomaterials field, R. A. Marklein *et al*. utilized this approach to crosslink a methacrylated hyaluronic acid system with both DTT and UV polymerization. The sliding opaque mask was used to control the radical polymerization in further stiffening the gels, achieving an average gradient of 6.5 kPa/mm and a stiffness range from ∼3 to ∼100 kPa over a 15 mm gel [22]. Kloxin *et al*. used a similar opaque mask to degrade PEG gels with photodegradable crosslinkers [23], obtaining an average gradient of 3 kPa/mm and a stiffness range from 10 to 30 kPa over a 9 mm gel. Here, we extend the moving mask technique to show that a higher gradient (up to 17.5-fold larger than [22] and 40-fold larger than [23]) and higher rigidity range (up to 3.4-fold larger than [22] and 12-fold larger than [23]) can be obtained by polymerizing an acrylamide/bis-acrylamide solution. Furthermore, we show that linear profiles can be obtained by moving the mask at a variable speed to balance the non-linear relationship between hydrogel polymerization and irradiation dose. Although the fabrication of *linear* gradient hydrogels requires varying mask speed, a constant mask displacement produces hydrogels with a steadily decreasing rigidity (Fig. 2B). For many applications, this will be a sufficiently good approximation to a linear profile.

Although the method presented here is suitable to produce linear stiffness gradients, we note that some non-linear stiffness profiles (e.g. exponential stiffness profiles) on the PAA hydrogels are hard to obtain. This difficulty possibly occurs because the photopolymerization of acrylamide depends not only on the amount of light received, but also on nucleation mechanisms of polymerization and perhaps also on the diffusion of the photoinitiator (Irgacure, in our case). This limitation may be overcomed by the use of another photocrosslinkable polymer, such as styrenated gelatin, which has been shown to polymerize in tight correlation with irradiation profile [24].

The method presented here allows us to reproduce most of the conditions observed *in vivo,* as the resulting hydrogels provide linear rigidity gradient profiles that cover most of the stiffness range physiologically relevant to cells. When we seed cells on our hydrogels, we observe that cell spreading correlates with substrate rigidity. This positive correlation has been extensively documented in various cell types [2] and has been used as a hallmark to quantify how cells sense the surrounding rigidity [3]. Our innovative hydrogels are particularly convenient for probing the spreading of cells under a wide range of rigidity conditions, as they provide a broad stiffness range. They also should be amenable to miniaturization. This technique provides a simple approach that produces steep gradients, wider rigidity ranges, and more accurate profiles than currently existing methods.

## Methods

### Fabrication of stiffness gradient matrices

Preparation of matrices is carried out by photopolymerization of an aqueous solution of acrylamide and bis acrylamide (Bio-Rad, Richmond, CA). Acrylamide concentrations varied between 8–15% and bis-acrylamide between 0.48–1%. To polymerize the solution, we add 0.5 mg/ml of Irgacure 2959 (BASF, Ludwigshafen, Germany), place the solution between two glass coverslips, one of them activated with 3-Aminopropyltriethoxysilane and glutaraldehyde. We irradiate the solution with a conventional ultraviolet (UV) lamp (Black Ray, 15W, 365 nm, UVP, Upland CA) held above the solution at a distance of 6 cm. The light flux delivered to the sample was 3.9 mW/cm^2^, as measured with a calibrated photodiode (PD-300-UV, Ophir Optronics Ltd., Israel). In general, the time needed to crosslink the acrylamide/bis-acrylamide solution depends on the amount of Irgacure used and the intensity of the UV irradiation, as well as its wavelength. The irradiation of the sample is minimally dimmed by the coverslip as we measured a 50% cut-off at 310 nm by the coverslip alone (Spectra Max Plus384, Molecular Devices, Silicon Valley, CA). The optimal Irgacure 2959 absorbance peak is 280 nm and, at 310 nm, the absorbance is 2/3 of the peak. So our use of a longer wavelength lamp slightly increases the exposure time needed to crosslink the solution. In our experimental setup, we found that 4 min was sufficient to crosslink the acrylamide/bis-acrylamide solution and obtain a hydrogel with stiffness of 240 kPa.

To obtain hydrogels having a stiffness gradient, we irradiate the acrylamide/bis-acrylamide solution with a spatially-varying light source. This light gradient is created by a programmable linear motion stage that progressively uncovers the acrylamide/bis-acrylamide solution by moving an opaque mask at a controlled speed [21] (Figs. 1A and B and Fig. S6). The resulting irradiation pattern creates a hydrogel with a Young's modulus (*E*) gradient that changes from 240 kPa in the most irradiated region to ∼1 kPa in the least irradiated one. The use of the sliding mask simplifies the fabrication setup, as there is no need for UV light source collimation. We use a conventional microscope stage (Proscan™, Prior Scientific Instruments Ltd, Cambridge, UK) to reach micrometer scale resolution in mask displacements. The irradiation is performed on an inverted microscope (IX70, Olympus, Japan) which enables us to image the irradiation profile while the mask is uncovering the acrylamide-bisacrylamide solution. Custom LabView™ software (National Instruments, Austin, TX) allows us to modulate the mask speed. Hydrogels are stored in water for two days prior to protein coating, to remove unreacted photoinitiator molecules.

### Design of the irradiation profile

Following irradiation of an acrylamide/bis-acrylamide solution, we found that the stiffness of the resulting hydrogel depends non-linearly on the total amount of light received (see Fig. 2B and later discussion). As a result, we needed to adjust the mask speed in order to obtain an irradiation profile that compensates for this effect. To determine the optimal mask speed variation, we first measured the stiffness of a hydrogel obtained by crosslinking the acrylamide/bis-acrylamide solution with a linear irradiation profile (Fig. 2C). Based on this information, we determined the irradiation sequence that produces a linear stiffness gradient hydrogel and the corresponding mask speed variation to generate it. These calculations were implemented using Matlab™ (The Mathworks, MA), as described in Text S1, Fig. S2 and Fig. S3. This procedure allows for the production of many irradiation profiles (see Fig. S2), although with PAA hydrogels the resulting stiffness profile may differ from the irradiation profile. Other polymer solutions may produce matrices with stiffness that more closely follow the irradiation profile (see Discussion).

### Characterization of stiffness gradient hydrogels by Atomic Force Microscopy

We characterize the hydrogel stiffness by the Young's modulus (*E*). Values of the spatially-dependent moduli of the stiffness-gradient hydrogels were measured by AFM, using a Bioscope Catalyst® NanoScope® V device (Bruker, Santa Barbara, CA) attached to an inverted optical microscope (IX71, Olympus, Japan). The gels were probed with a V-shaped cantilever (MSCT, pyramidal tipped, nominal *k* = 0.03 N/m; Bruker) whose spring constant was calibrated by the thermal fluctuations method [25], [26]. The relationship between photodiode signal and cantilever deflection was computed from the slope of the force displacement curve obtained at a bare region of the coverslip (i.e., outside the gel sample). For each gel point, we acquired ten force-displacement (*F*-*z*) curves (where *F* = *kd*, *d* being the deflection of the cantilever) by monitoring *F* and *z* while the piezo translator was ramped forward and backward at constant speed (5 µm amplitude, 1 Hz and ∼1 µm of indentation, less than the tip height which is 2.5 µm). Each experimental *F*-*z* curve was fitted to the four-sided pyramidal indenter model [18]:
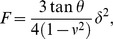
where *E* is the Young's modulus,*ν* is the Poisson's ratio, θ is de semiincluded angle of the pyramidal indenter, and *δ* is the indentation depth. The parameter *ν* is assumed to be 0.5 (the water-filled hydrogel essentially is incompressible), and the indentation depth is calculated as *δ* = *z*−*z*_0_−*d*, where *z*_0_ is the tip-gel contact point. *E* and *z*_0_ were estimated by least-squares fit of this equation to the *F*-*z* curve recorded on each gel point [27]. We verified the hydrogel mechanical measurements using hydrogels of known stiffness, as described in Ref. [28]. For hydrogel regions of stiffness <1 kPa, we were unable to provide a reliable measurement of *E*. However, in our hydrogels, this part corresponded to a small region of <50 µm away from the hydrogel border. We measured the Young's modulus of the gel every 200–300 µm (∼5 times the length of a mammalian cell) along the axis of maximum gel stiffness change.

### Hydrogel protein coating and characterization

Fibronectin was covalently grafted to the polymerized hydrogels through free amino groups by succinimide chemistry [16], [17]. Aliquots of Sulfo-SANPAH were prepared by dissolving Sulfo-SANPAH (Pierce Biotechnology, Rockford, IL) in anhydrous dimethyl sulfoxide (DMSO) (20 µl/mg of Sulfo-SANPAH). Stocks were frozen on dry ice and stored at −80°C for later use. Sulfo-SANPAH-DMSO aliquots were diluted in double-distilled water (2 mg/ml, pH 7) immediately before use to coat a gel surface (∼60 µl/cm^2^). Due to the short half-life of Sulfo-SANPAH in water, these steps were done in less than 2 minutes. The hydrogels were irradiated under the UV light for 3 min and washed thoroughly with distilled water. A 20 µl drop of 0.1 mg/ml fibronectin (in PBS pH 7.4, isolated from human plasma, Sigma-Aldrich, St Louis, MO) was placed in parafilm sheet. The sulfo-SANPAH activated hydrogel was inverted on top of the fibronectin drop and incubated at room temperature for 2 h (∼2.5 µg fibronectin/cm^2^). Previous work has shown that incubation of 0.1 mg/ml protein solutions for more than 30 min is sufficient to produce saturating levels of adhesion proteins on hydrogels independent of their substrate stiffness [3]. After the incubation, hydrogels were extensively washed with PBS and incubated for at least 1 h with cell media prior to seeding cells.

We used immunofluorescence to confirm that the density of fibronectin on the hydrogel surface did not depend on gel stiffness and does not display obvious heterogeneity. Three typical gels, prepared as discussed above, were rinsed once in TBS (Tris buffered saline: 50 mM Tris, 150 mM NaCl, pH 8.4), and incubated with anti-fibronectin primary antibody (1∶500, rabbit polyclonal, F3648, Sigma-Aldrich) for 1 h at room temperature. The gels were then rinsed five times in TBS and incubated in secondary antibodies (1∶1000, Rhodamine anti-rabbit, Jackson ImmunoResearch Laboratories, West Grove, PA). The resulting fluorescently stained hydrogels were imaged using an Olympus IX70 inverted wide-field microscope equipped with a mercury vapor lamp and a Hamamatsu C9100 camera. In addition, confocal cross-sectional fluorescence images were obtained with a Zeiss LSM 510 META microscope.

### Cell culture

Cell behavior on fabricated stiffness gradient hydrogels was tested principally with NIH3T3 fibroblasts, though some experiments used SY5Y neuroblastoma cells. The NIH3T3 cell line was a gift from Dr. Suresh Ambudkar, NCI/NIH [29]. The cell line identity as NIH3T3 was confirmed by PCR and micro-satellite analysis performed by Idexx Radil, Columbia MO. The SY5Y cell line was obtained from Dr. June Biedler (Memorial Sloan-Kettering Cancer Center), the originator of the line [30], and was a kind gift from Dr. Carol Thiele, NCI/NIH. Cells were cultured in RPMI 1640 medium supplemented with 1 mM L-glutamine (Cell-Gro, Manassas, VA), 100 U ml^−1^ penicillin, 100 mg ml^−1^ streptomycin (both from Life Technologies, Carlsbad, CA) and 10% fetal bovine serum (Thermo Scientific, Waltham, MA). Cells were incubated at 37°C and 5% CO_2_. Two days before the experiments, cells were harvested by means of a brief exposure to trypsin EDTA (Life Technologies) and plated sparsely on 22 mm diameter glass cover slips (30 cells mm^−2^) upon which a stiffness gradient gel coated with fibronectin had been formed. After 20 h of incubation, cells were observed with phase contrast under the microscope. The public domain software ImageJ (NIH, Bethesda, MD), along with the plug-in MosaicJ, was used to quantify cell spreading area. A minimum of 400 cells were used to quantify area variation as a function of substrate stiffness.

## Supporting Information

Text S1 **Controlling the irradiation profile by varying mask speed.**
(PDF)

Figure S1 **Mask speed can modulate the hydrogel elasticity slope while maintaining a comparable stiffness range.** Spatial map of elasticity of hydrogels obtained with the same acrylamide/bisacrylamide/Irgacure solution but different mask speeds. Hydrogels obtained with a mask speed of 7.5 µm/s (green line) resulted in a gradient slope of 170 kPa/mm. Hydrogels obtained with a mask speed of 30 µm/s (red line) displayed a gradient slope of 90 kPa/mm. Finally, hydrogels obtained with 100 µm/s (blue line) showed a slope that changed from 50 to 17 kPa/mm. Error bars in each hydrogel represent SE of 3 replicates.
(TIF)

Figure S2 **Examples of irradiation profiles obtained by moving the mask using different speed protocols.** The method presented here is suitable for obtaining linear (A), exponential (B) and general monotonically decreasing (C) irradiation profiles. Dashed line in panels A–C indicates the initial position of the mask. These data have been obtained by imaging the real movement of the mask and then summing all recorded frames. Bottom panels display the mask speed protocol used to obtain the linear (D), the exponential (E) and the general monotonically decreasing (F) irradiation profiles shown in A–C panels.
(TIF)

Figure S3 **Correction for non-linear relation between irradiation and stiffness.** (A) From the data in the calibration curve (Fig. 2C), we ascertain the irradiation pattern (solid line) that produces a linear stiffness hydrogel (dashed line). (B) Sketch representing the moving mask setup at time *t*. The edge of the mask is positioned at *x*(*t*) = *x* and is moved at a speed *v*(*t*) = *v*. *L* is the hydrogel length and *T* is the maximum irradiation time (i.e. *T* is the time that needs the edge of the mask to travel from *x* = 0 to *x* = *L*). The exposure time of the hydrogel at position *x* is given by *T* – *t_x_*, where *t_x_* is the time at which the mask arrives at gel position *x*.
(TIF)

Figure S4 **Hydrogel stiffness profiles are reproducible. Spatial map of elasticity of hydrogels produced on different days with different solutions of acrylamide/bis-acrylamide/Irgacure.**
(TIF)

Figure S5 **Hydrogels treated with Sulfo-SANPAH incorporate substantially more fibronectin (FN) than untreated ones.** Fluorescence intensity profile along a gradient hydrogel using the Sulfo-SANPAH protocol described in the Methods Section (blue squares), incubation with fibronectin in absence of sulfo-SANPAH (red squares), or incubation with BSA alone. Error bars represent SE of 3 replicates.
(TIF)

Figure S6 **Stiffness gradient fabrication schematic.** (A) The acrylamide/bis-acrylamide/Irgacure solution is placed between 2 coverslips and supported on the top of a 4× objective by means of a holder. The mask is attached to a microscope stage that allows precise control of the mask speed. The sample is illuminated by a UV bench lamp placed on the top of the setup. (B) The holder is an empty cylinder that allows one to image the solution as the mask progressively uncovers the polymerizing solution.
(TIF)
