# Supplementary material for: Fabrication of Hydrogels with Steep Stiffness Gradients for Studying Cell Mechanical Response
Source: PLoS One. 2012 Oct 4;7(10):e46107. doi: 10.1371/journal.pone.0046107 (PMC3464269; doi:10.1371/journal.pone.0046107)
Supplement: Text S1 — Controlling the irradiation profile by varying mask speed. (PDF) [file pone.0046107.s001.pdf]

Fabrication of hydrogels with steep stiffness gradients for studying cell mechanical response

Raimon Sunyer, Albert J. Jin, Ralph Nossal and Dan L. Sackett

Supporting Text S1

Text 1. ***Controlling the irradiation profile by varying mask speed***

When we expose an acrylamide/bis-acrylamide/Irgacure solution with a linear gradient of irradiation, a non-linear stiffness profile is produced (Fig. 2B). We need to adjust the mask speed to obtain an irradiation profile that makes up for this non-linear relationship between stiffness and irradiation. In principle, varying the mask speed during the hydrogel polymerization process allows us to generate any desired monotonically decreasing irradiation pattern (Fig. S2). The procedure to linearize the stiffness gradient hydrogels consists of 2 steps. First, the calibration curve (Fig. 2C) is used to convert the desired linear stiffness profile, $E(x)$, to the corresponding irradiation dose profile. Second, the mask speed protocol is calculated that reproduces the irradiation dose profile.

Determination of the irradiation dose profile

Interpolating the calibration curve (Fig. 2C), we establish the irradiation dose profile that produces the desired linear stiffness gradient profile, $E(x)$, of the hydrogel (Fig. S3A). This irradiation dose profile will have units of mJ/cm^2 and will monotonically decrease over the hydrogel position x. The exposure time at each hydrogel position, $\tau(x)$, will be obtained by dividing the irradiation dose profile by the UV lamp power.

Determination of the speed protocol that produces the irradiation dose profile

We use the exposure time at each hydrogel position, $\tau(x)$, to calculate the speed protocol that produces $E(x)$. Fig. S3B depicts the mask setup at time t, when the edge of the mask is positioned at $x(t) = x$ and is moving with a speed $v(t) = v$. Let T represent the maximum exposure time needed to polymerize the region of maximal stiffness, which is equivalent to the time required for the edge of the mask to travel from $x = 0$ to $x = L$, where L is the length of the gel. Then, the total exposure time of the hydrogel at position x is given by $T-t_x$, where t_x is the time at which the mask arrives at x. For each hydrogel position, we equate the total exposure time of the hydrogel with the corresponding desired exposure time:

$$T - t_x = \tau(x). \qquad \textbf{Eq. 1}$$

The mask speed protocol as a function of time, $v(t_x)$, can be obtained by expressing x as a function of t_x in Eq. 1 and calculating its derivative. In general, for complex forms of the desired exposure time $\tau(x)$ (see, for illustration, Fig. S3A), one needs to numerically solve Eq. 1. For the irradiation dose profiles used to create the gels discussed in this paper, such calculations were implemented using computer code written in Matlab™ (The Mathworks, MA). This program is available from Dr. R. Sunyer upon request.
