# Supplementary figures and images for: Fabrication of Hydrogels with Steep Stiffness Gradients for Studying Cell Mechanical Response
Source: PLoS One. 2012 Oct 4;7(10):e46107. doi: 10.1371/journal.pone.0046107 (PMC3464269; doi:10.1371/journal.pone.0046107)

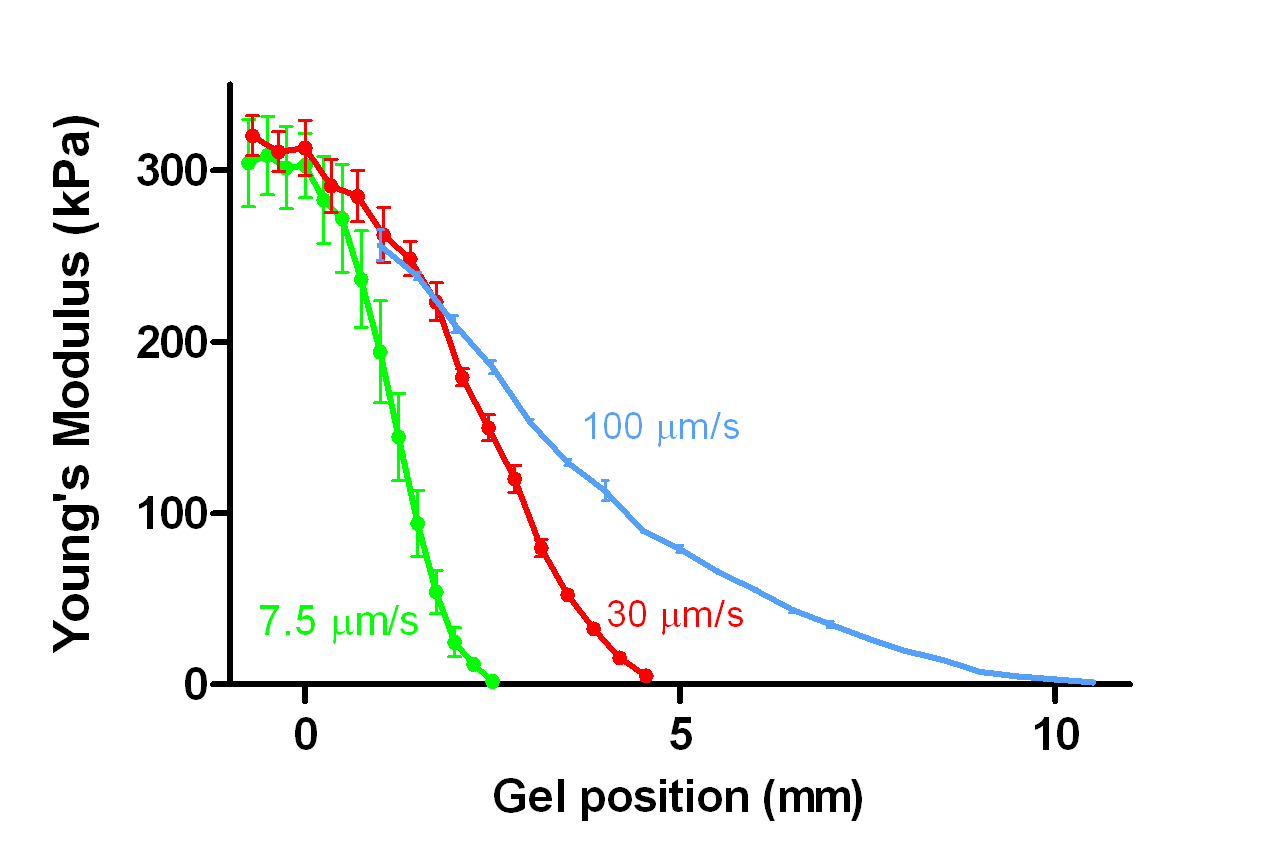

Supplement: Figure S1 — Mask speed can modulate the hydrogel elasticity slope while maintaining a comparable stiffness range. Spatial map of elasticity of hydrogels obtained with the same acrylamide/bisacrylamide/Irgacure solution but different mask speeds. Hydrogels obtained with a mask speed of 7.5 µm/s (green line) resulted in a gradient slope of 170 kPa/mm. Hydrogels obtained with a mask speed of 30 µm/s (red line) displayed a gradient slope of 90 kPa/mm. Finally, hydrogels obtained with 100 µm/s (blue line) showed a slope that changed from 50 to 17 kPa/mm. Error bars in each hydrogel represent SE of 3 replicates. (TIF) [file pone.0046107.s002.tif]

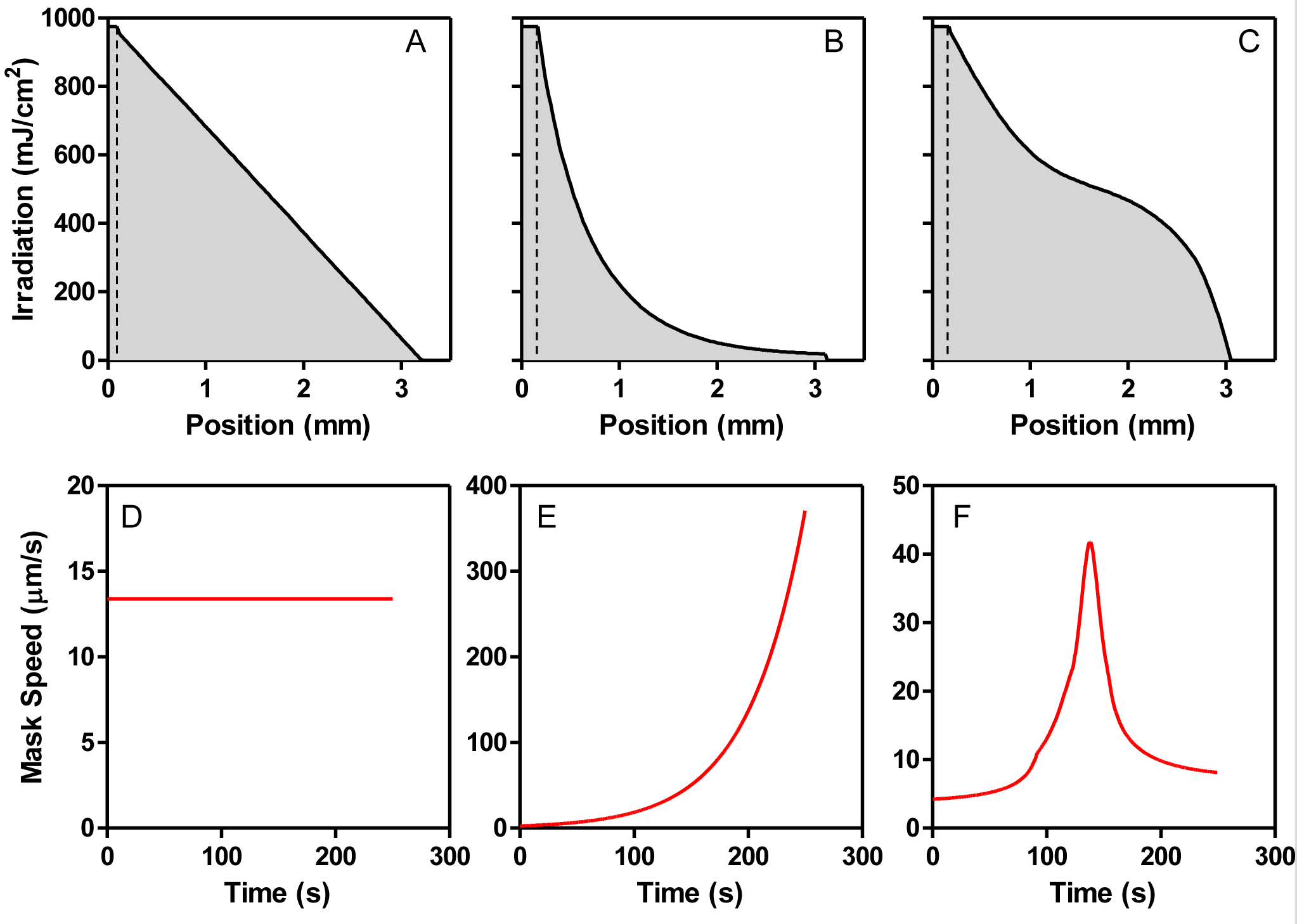

Supplement: Figure S2 — Examples of irradiation profiles obtained by moving the mask using different speed protocols. The method presented here is suitable for obtaining linear (A), exponential (B) and general monotonically decreasing (C) irradiation profiles. Dashed line in panels A–C indicates the initial position of the mask. These data have been obtained by imaging the real movement of the mask and then summing all recorded frames. Bottom panels display the mask speed protocol used to obtain the linear (D), the exponential (E) and the general monotonically decreasing (F) irradiation profiles shown in A–C panels. (TIF) [file pone.0046107.s003.tif]

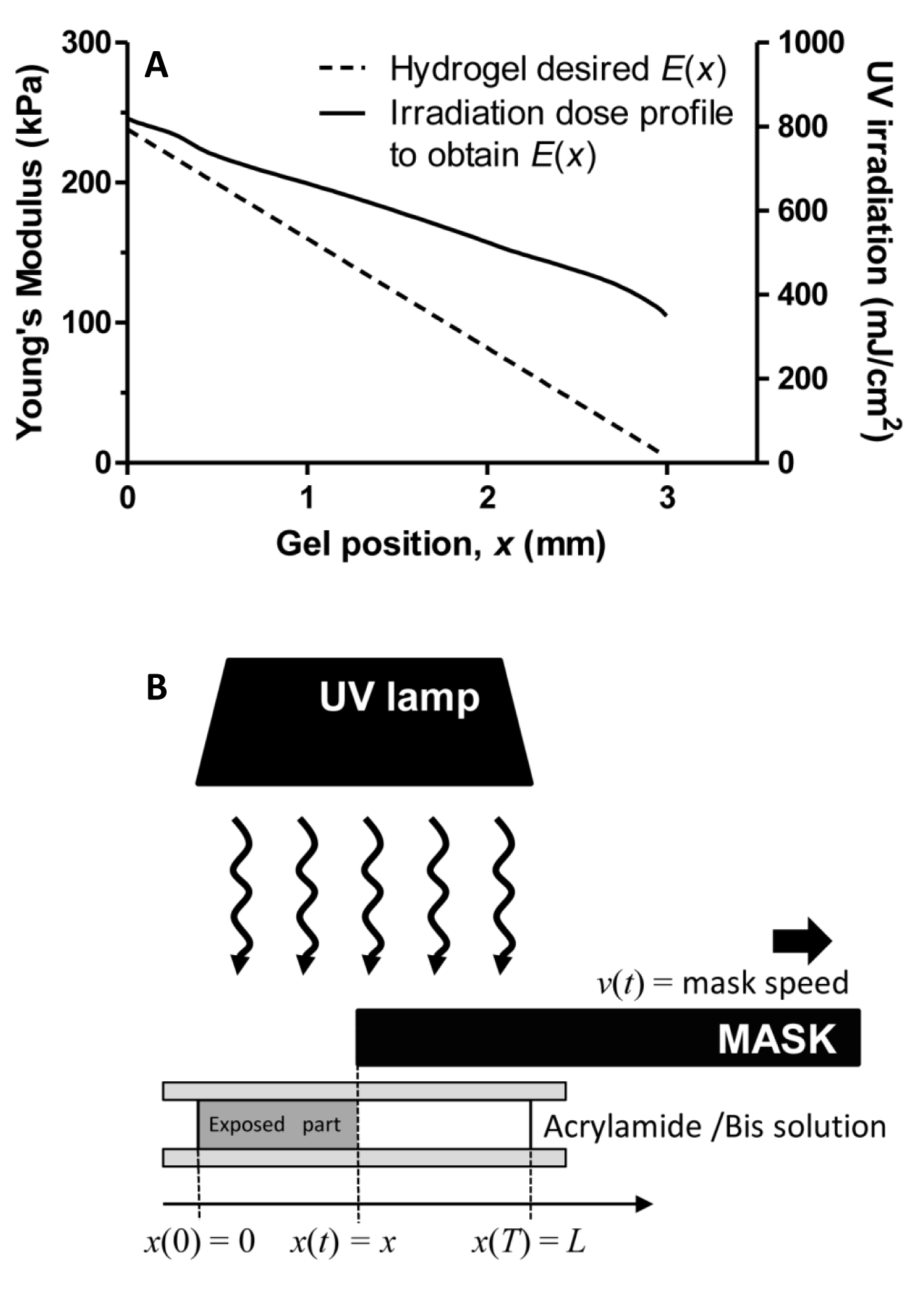

Supplement: Figure S3 — Correction for non-linear relation between irradiation and stiffness. (A) From the data in the calibration curve (Fig. 2C), we ascertain the irradiation pattern (solid line) that produces a linear stiffness hydrogel (dashed line). (B) Sketch representing the moving mask setup at time t. The edge of the mask is positioned at x(t) = x and is moved at a speed v(t) = v. L is the hydrogel length and T is the maximum irradiation time (i.e. T is the time that needs the edge of the mask to travel from x = 0 to x = L). The exposure time of the hydrogel at position x is given by T – tx, where tx is the time at which the mask arrives at gel position x. (TIF) [file pone.0046107.s004.tif]

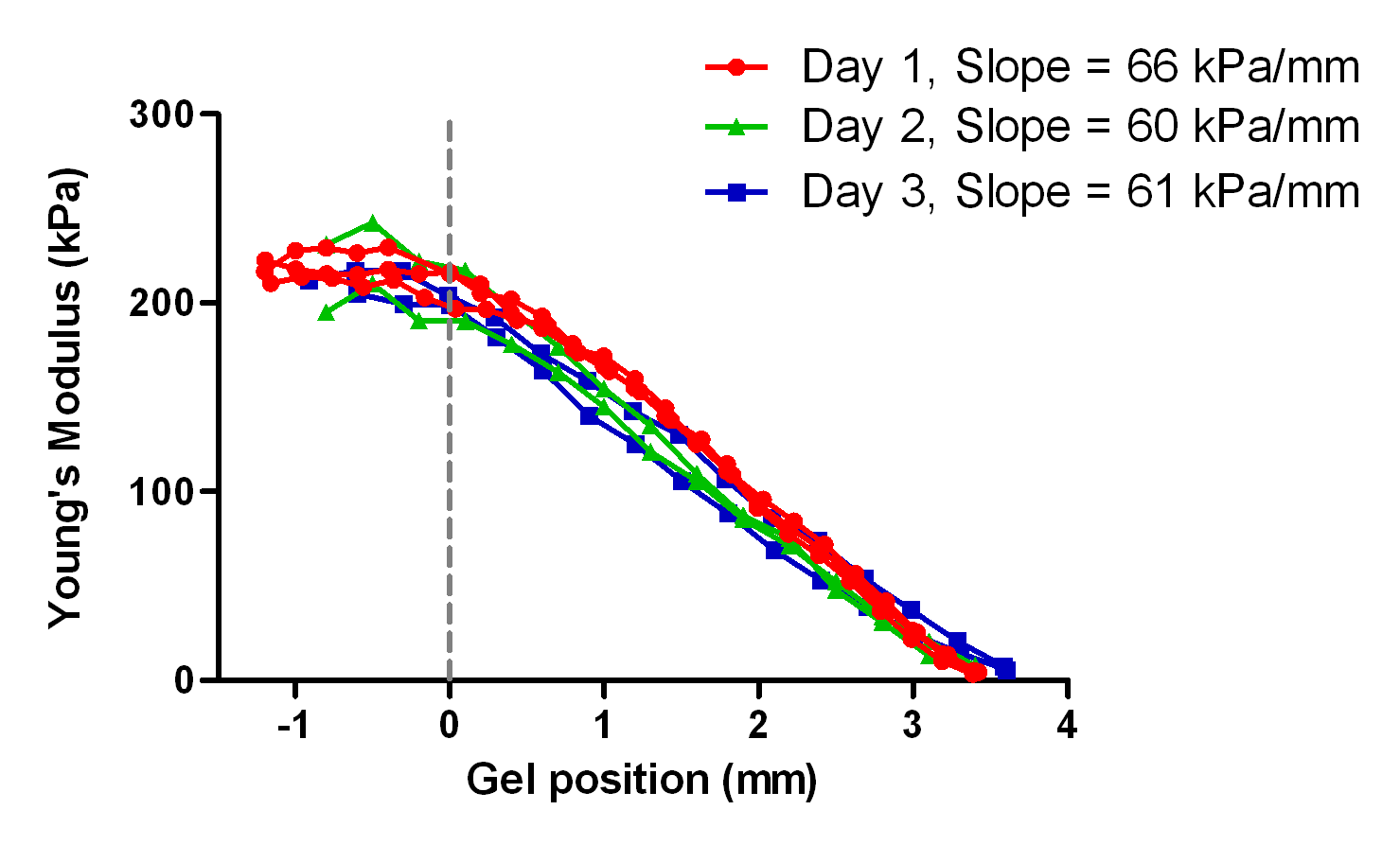

Supplement: Figure S4 — Hydrogel stiffness profiles are reproducible. Spatial map of elasticity of hydrogels produced on different days with different solutions of acrylamide/bis-acrylamide/Irgacure. (TIF) [file pone.0046107.s005.tif]

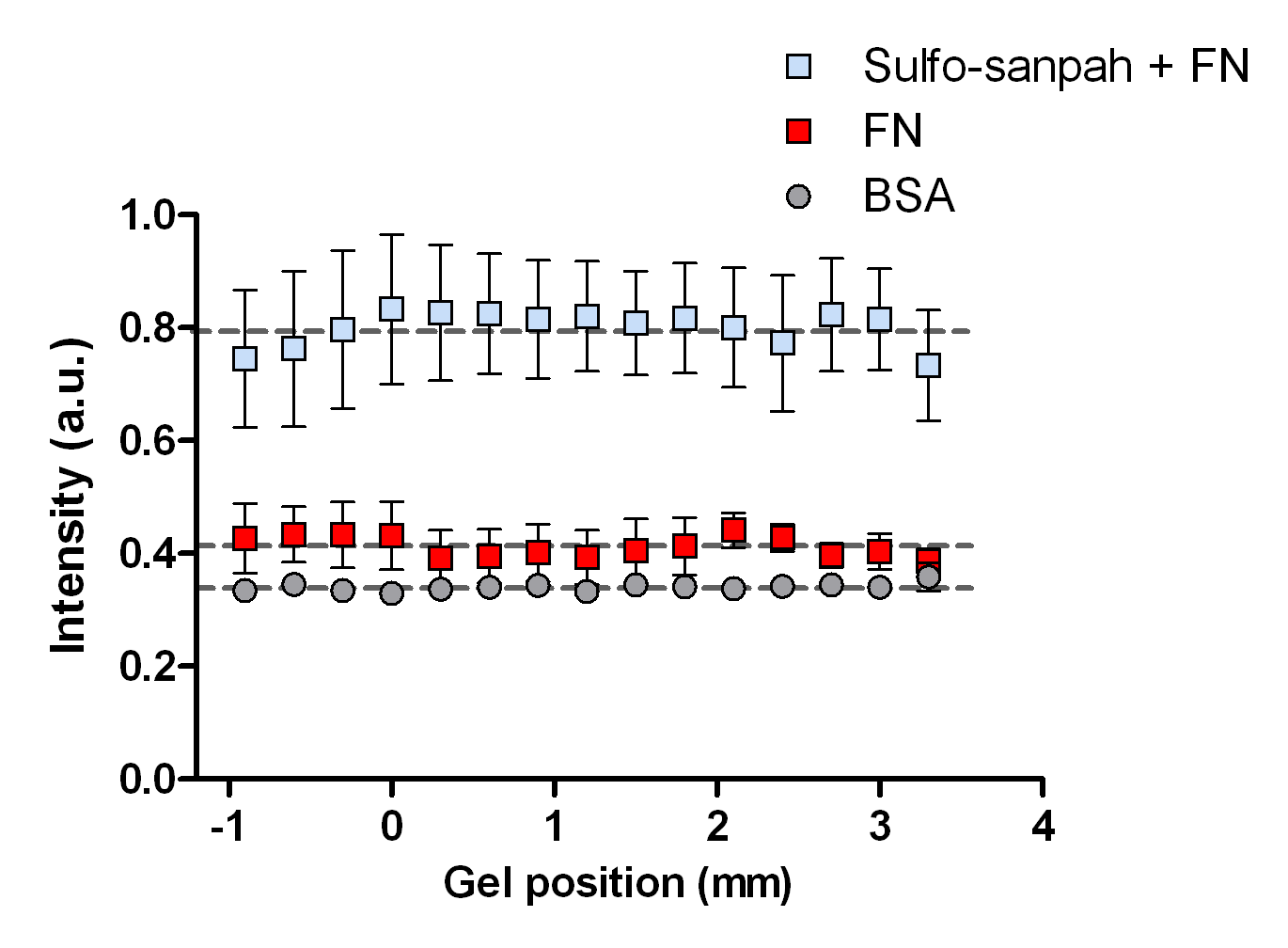

Supplement: Figure S5 — Hydrogels treated with Sulfo-SANPAH incorporate substantially more fibronectin (FN) than untreated ones. Fluorescence intensity profile along a gradient hydrogel using the Sulfo-SANPAH protocol described in the Methods Section (blue squares), incubation with fibronectin in absence of sulfo-SANPAH (red squares), or incubation with BSA alone. Error bars represent SE of 3 replicates. (TIF) [file pone.0046107.s006.tif]

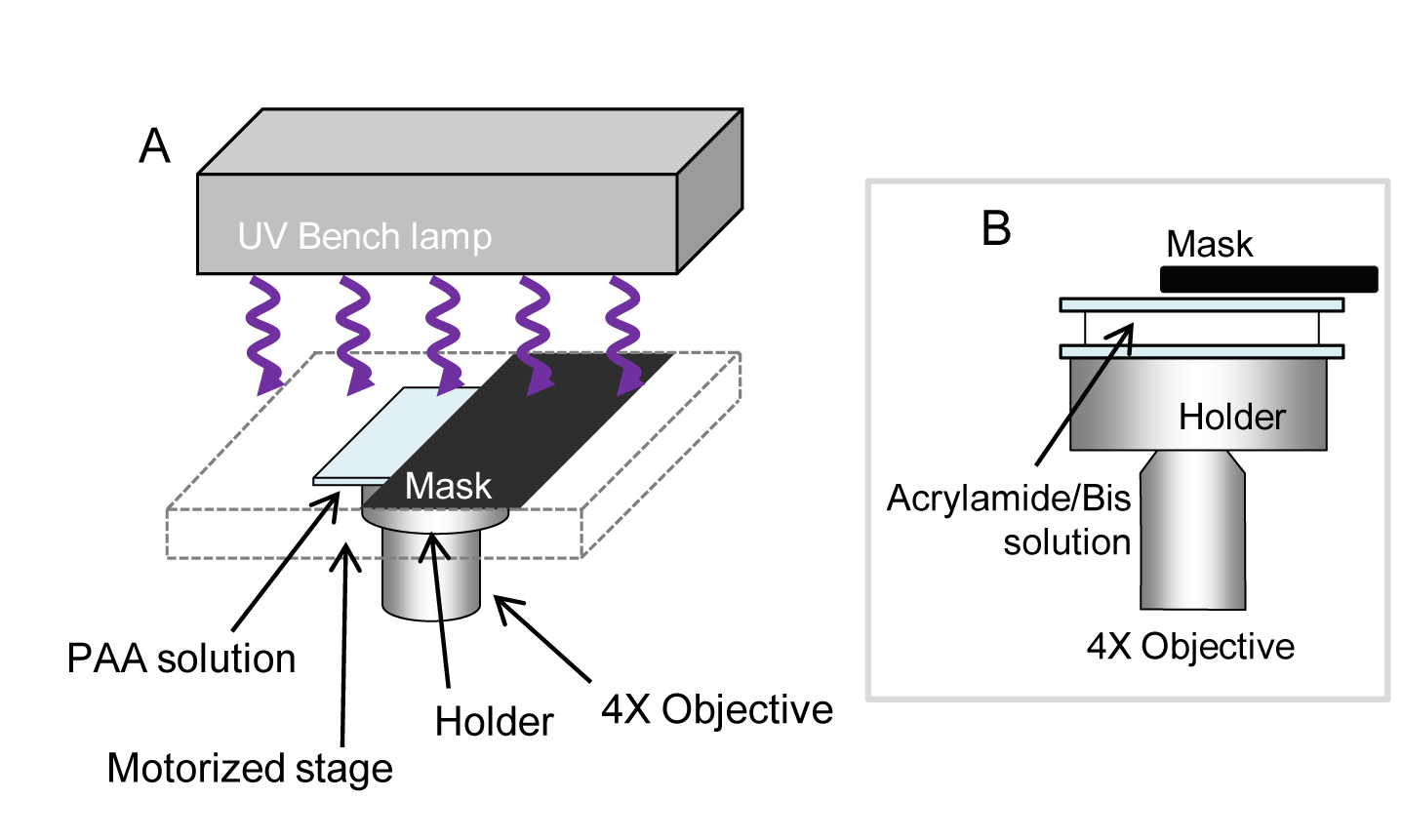

Supplement: Figure S6 — Stiffness gradient fabrication schematic. (A) The acrylamide/bis-acrylamide/Irgacure solution is placed between 2 coverslips and supported on the top of a 4× objective by means of a holder. The mask is attached to a microscope stage that allows precise control of the mask speed. The sample is illuminated by a UV bench lamp placed on the top of the setup. (B) The holder is an empty cylinder that allows one to image the solution as the mask progressively uncovers the polymerizing solution. (TIF) [file pone.0046107.s007.tif]
